# Phylogeny, phylogeography, and conservation of a rediscovered gecko from the Galápagos Islands

**DOI:** 10.1371/journal.pone.0324659

**Published:** 2025-06-13

**Authors:** Omar Torres-Carvajal, Paula A. Castaño, Enrique Rincón, Fernando Ayala-Varela, Karl Campbell, Wilson Cabrera, Francisco Moreno

**Affiliations:** 1 Museo de Zoología, Biología, Pontificia Universidad Católica del Ecuador, Quito, Ecuador; 2 Island Conservation, Charles Darwin Ave, Puerto Ayora, Galápagos Islands, Ecuador; 3 Re:wild, Charles Darwin Avenue, Puerto Ayora, Galápagos Islands, Ecuador; 4 Dirección del Parque Nacional Galápagos, Charles Darwin Avenue, Puerto Ayora, Galápagos Islands, Ecuador; L3 Scientific Solutions, GERMANY

## Abstract

The rediscovery of species in iconic conservation sites like the Galápagos is eye-catching and can lead to quick and effective conservation actions. With 11 species occurring on most islands, Galápagos leaf-toed geckos (*Phyllodactylus*) are among the least known terrestrial vertebrates of the archipelago. Strikingly, reliable records of *Phyllodactylus* from Rábida Island prior to this study are limited to Holocene subfossils and a single photograph from 2012. Here we report the first vouchered specimens of *Phyllodactylus* from Rábida and present their phylogenetic and phylogeographic affinities with other gecko populations in the archipelago. Despite taxonomic uncertainty, we recognize Rábida gecko populations as a separate ESU within *P. maresi*, which also occurs in nearby islands (Santiago, Bartolomé, Mares), as well as in Marchena. Finally, we suggest that Rábida gecko populations benefited from the eradication of invasive rodents, which facilitated their rediscovery and the collection efforts reported here.

## Introduction

The rediscovery of species previously thought to be extinct has received considerable attention in recent years [1,2]. Some of the factors influencing rediscovery are (1) range and population size, (2) distribution within tropical megadiverse countries, (3) incorrect declaration of “extinct” status, (4) lifeform (plants), (5) accessibility to habitats of lost species, (6) species’ charisma, and (7) search effort [1–4]. The rediscovery of species in iconic conservation areas, such as the Galápagos Islands, attracts special attention and can result in quick conservation actions. For example, soon after the rediscovery in 2019 of the giant tortoise *Chelonoidis phantasticus* in Fernandina Island, the only female found, Fernanda, was transferred to the Galápagos National Park Tortoise Center, and further expeditions aimed at finding males for captive breeding were initiated [5].

In contrast to the charismatic giant tortoises, many Galápagos organisms have received less attention. Due to their secretive habits and nocturnal activity, leaf-toed geckos (*Phyllodactylus*) are perhaps the least known terrestrial vertebrates from the Galápagos Archipelago. Their present diversity stems from three colonization events originating in South America [6]. Of these, two events (~0.69 Mya and ~3.03 Mya) led to a single extant species (*Phyllodactylus giberti* in Wolf Island and *P. darwini* in San Cristobal Island, respectively). The third and oldest radiation (*P. galapagensis* radiation), which started at ~5.49 Mya, resulted in nine species across most of the islands [7,8]. Interestingly, despite intense collecting efforts in the Galápagos, especially in the late 19^th^ and early 20^th^ centuries [9–11], no geckos were registered from one of its main islands, Rábida (a.k.a. Jervis). This 499-ha island emerged between 1.3–1.6 million years ago [12] and lies in the central part of the archipelago, about 4.5 km south of Santiago Island and 25 km east of Isabela Island. Prior to 2012, the only record of geckos from this island were Holocene subfossil bones (5,700–8,540 years B. P.) reported by Steadman et al. [13], who also mentioned that “the current absence on Rábida of the secretive *Phyllodactylus* may only be an artifact of inadequate collecting.” Subsequently, in their field guide to Galápagos vertebrates, Swash and Still [14] reported *Phyllodactylus* from Rábida as an undescribed, possibly extinct species without providing further details. Thus, leaf-toed geckos in Rábida were suspected of having gone extinct prior to humans discovering the archipelago.

In 2011, the Galápagos National Park and its conservation partners successfully implemented an invasive rodent eradication program targeting brown rats (*Rattus norvegicus*) [15]. Shortly after removing invasive rodents, a team of researchers and Galápagos National Park rangers monitoring Rábida Island in 2012 found a specimen of *Phyllodactylus* [15]. This specimen was collected and photographed, thus representing the first record in the literature of a living leaf-toed gecko from Rábida Island. Subsequent reports of *Phyllodactylus* from Rábida by other authors [14,16] are not reportedly based on vouchers, photographs or even sightings. In this paper, we report on the rediscovery of leaf-toed geckos from Rábida based on two recent expeditions that allowed us to collect both tissue samples and specimens. We present for the first time their phylogenetic and phylogeographic affinities to other gecko populations from the Galápagos based on analyses of DNA sequence data, as well as a general morphological characterization and comparison with related taxa.

## Materials and methods

### Fieldwork and data sampling

Samples were obtained during two expeditions to Rábida Island. In October 2019, we sampled muscle tissue (tail tips) from nine specimens of leaf-toed geckos from Rábida and stored those samples in Longmire buffer [17] before releasing the specimens. These tissue samples were deposited at Museo de Zoología QCAZ, Pontificia Universidad Católica del Ecuador, Quito (QCAZ 17562–570). In a second expedition between 5–7 August 2021, we collected 10 whole body gecko specimens, which were captured by hand between 18:00–23:00 h and photographed the next morning. Most specimens were active on lava rocks. After lethal anesthetization with an intracelomic injection of benzocaine (2%), muscle and liver tissue samples were extracted and stored in Longmire buffer. Specimens were then fixated in 10% formalin and stored in 70% ethanol in the Vertebrate Collection of the Charles Darwin Foundation in Puerto Ayora, Galápagos (VCCDRS 3415–3424). This study was evaluated and approved by the DINV (Dirección de Investigación) of the Pontificia Universidad Católica del Ecuador in accordance with the guidelines for environmental and social impacts of research projects. The DINV committee evaluates projects to determine observance of its norms for ethical scientific research. Genetic data were obtained under the Genetic Resources Access Contract No MAE-DNB-CM-2016–0060 issued by the Ecuadorian Ministry of Environment, Water, and Ecological Transition. Science permit PC-56–23 and export permits 159–2019 DPNG and 070–2023 DPNG were issued by the Galápagos National Park Directorate (DPNG).

### DNA sequence data and phylogenetic analyses

Tissue samples were mixed with Proteinase K and lysis buffer before overnight digestion. Total genomic DNA was extracted following a guanidinium isothiocyanate extraction protocol. DNA samples were quantified with a Nanodrop® ND-1000 (NanoDrop Technologies, Inc.), re-suspended and diluted to 25 ng/ul in ddH_2_O prior to amplification. We used the same primers and amplification protocols described in Torres-Carvajal *et al.* [8].

We obtained new DNA sequence data from five samples of *Phyllodactylus* (QCAZ 17562–566) from Rábida. We combined those sequences with 130 GenBank sequences of 23 species of *Phyllodactylus* from Galápagos and continental South America [6,8,18]. The species *Phyllodactylus nocticolus*, *P. unctus* and *P. xanti* were used as outgroups to root the tree [8]. We analyzed a character matrix of 135 terminals and 5,086 aligned nucleotides (nt) encompassing four mitochondrial and six nuclear genes. Mitochondrial genes included the NADH dehydrogenase subunit 4 (*ND4*, 601 nt) and a continuous fragment encompassing *12S* rRNA, *tRNA*^Val^, and *16S* rRNA (1,995 nt). Nuclear genes included brain-derived neurotrophic factor (*BDNF*, 630 nt), oocyte maturation factor MOS (*CMOS*, 390 nt), recombination-activating gene 1 (*RAG1*, 304 nt), recombination-activating gene 2 (*RAG2*, 384 nt), acetylcholinergic receptor M4 (*ACM4*, 383 nt) and phosducin (*PDC*, 399 nt). Sequences generated in this study are available on GenBank (Table 1). Although we were not able to obtain new sequences for *ND4*, we included this gene in the analyses because it is phylogenetically informative [8] and available in GenBank for most taxa included in our analyses.

**Table 1:** GenBank accession numbers for sequences obtained in this study.

Taxon	Voucher	Lat.	Long.	GenBank accession number							
				*12S- tRNA* ^*Val*^	*16S*	*ACM4*	*BDNF*	*CMOS*	*PDC*	*RAG1*	*RAG2*
*P. maresi*	QCAZ17562	−0.401	−90.706	PV437217	PV437249	PV443914	PV443918	PV443922	PV443927	PV443932	PV443937
*P. maresi*	QCAZ17563	−0.402	−90.707	PV437218	PV437250	PV443915	—	PV443923	PV443928	PV443933	PV443938
*P. maresi*	QCAZ17564	−0.402	−90.707	PV437219	PV437251	PV443916	PV443919	PV443924	PV443929	PV443934	—
*P. maresi*	QCAZ17565	−0.401	−90.706	PV437220	PV437252	—	PV443920	PV443925	PV443930	PV443935	PV443939
*P. maresi*	QCAZ17566	−0.399	−90.706	PV437221	PV437253	PV443917	PV443921	PV443926	PV443931	PV443936	PV443940

All samples were collected in Ecuador, Galápagos, Rábida Island. QCAZ: Museo de Zoología, Pontificia Universidad Católica del Ecuador, Quito, Ecuador.

We assembled and aligned DNA sequences in GENEIOUS PRIME 2022.1. (https://www.geneious.com) under default settings for MAFFT [19]. We translated all protein-coding sequences into amino acids for confirmation of alignment. In addition, we realigned ribosomal gene regions with multiple gaps in an attempt to minimize indels and optimize nucleotide identities across different individuals.

Phylogenetic relationships were inferred under the maximum likelihood optimality criterion in IQ-TREE [20]. We used MODELFINDER [21] to infer models of evolution for each gene, except for the *tRNA*^Val^ gene, which was included in the same partition as *12S* [8]. Branch support was also assessed in IQTREE with 1,000 replicates under both ultrafast bootstrapping [22] and SH approximate likelihood ratio test (SH-aLRT) [23]. We also ran a partitioned Bayesian analysis in MRBAYES v3.2.7 [24], with all parameters unlinked between partitions (except topology and branch lengths) and rate variation (prset ratepr = variable) invoked. We performed four independent runs of 5 million generations, each with four MCMC chains, sampling every 1,000 generations. After verifying convergence and effective sample sizes (≥ 200) in TRACER v1.7 [25], we discarded the initial 500 trees per run as “burn-in” before calculating posterior probabilities on a consensus tree.

### Phylogeography

To investigate haplotype diversity between leaf-toed geckos from Rábida and other related populations, we calculated haplotype networks for mitochondrial genes *12S* and *16S* using the software PopArt [26]. Because sequences of both *12S* and *16S* were either incomplete or not available for some samples, we trimmed individual *12S* (n = 16, 315 nt) and *16S* (n = 12, 1,259 nt) matrices until all sequences had < 5% of missing data. We also analyzed a concatenated *12S-16S* dataset after removing samples missing either *12S* or *16S* sequences (n = 11, 1,574 nt). We used the median-joining haplotype network algorithm, with parameter epsilon set to 0 [27]. We also addressed genetic differentiation within the *Phyllodactylus galapagensis* radiation [8] by calculating uncorrected genetic distances for *12S* and *16S* in PhyML 3.3 [23] using DIVEIN [28].

### Morphological data

We studied external morphology in all specimens obtained in the 2021 expedition and used published data [11] for comparisons with closely related taxa. We followed terminology for measurements and scutellation from the literature [11,29,30] to record the following characters: (1) number of scales between nostril and eye including the one or two scales on anterior margin of preorbital groove; (2) number of scales across snout at level of third labials; (3) number of scales across interorbital region at level of center of eyes; (4) number of supralabials from rostral to a point below center of eye; (5) number of infralabials from mental to a point below center of eye; (6) number of postmentals; (7) number of scales in contact with postmentals posteriorly; (8) number of scales in contact with scales behind postmentals; (9) number of longitudinal rows of dorsal tubercles at midbody; (10) number of dorsal tubercles counted on the medialmost (paravertebral) row from head to base of tail; (11) number of paravertebral tubercles between axilla and groin; (12) number of scales around midbody; (13) number of lamellae on Finger IV; (14) number of lamellae on Toe IV; (15) eye diameter; (16) head length between snout and anterior margin of ear opening; (17) snout length between snout and anterior margin of orbit; (18) length between axilla and groin; (19) snout–vent length (SVL); and (20) tail length (TL). SVL and TL measurements were taken with a ruler and recorded to the nearest millimeter. Other measurements were made with digital calipers and recorded to the nearest 0.1 mm. Sex was determined by gonad inspection through dissection, or by noting the presence or absence of (1) hemipenes by eversion or (2) shelled eggs visible through the skin.

## Results

**Fig 1:**
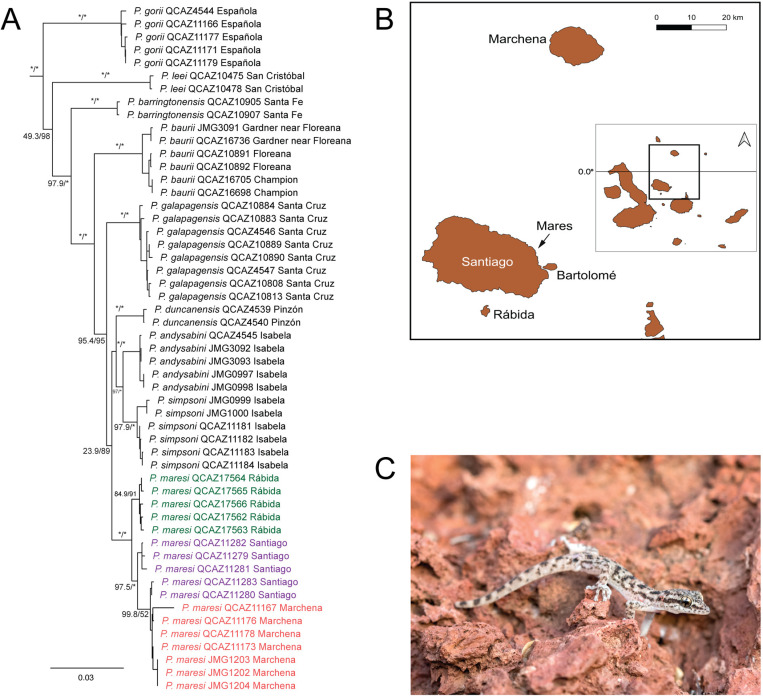
Phylogenetic relationships of leaf-toed geckos (*Phyllodactylus*) from Rábida, Galápagos Islands. **A.** Maximum-likelihood phylogenetic tree of leaf-toed geckos (*Phyllodactylus*) from Galápagos. Only the *P. galapagensis* radiation (i.e., all Galapagean species except *P. darwini*-San Cristóbal and *P. gilberti*-Wolf) is shown for clarity; the full tree is shown in S2 Fig. New samples from Rábida are shown in green. Numbers next to branches are SH-aLRT/ultrafast bootstrap support values, with asterisks representing values ≥ 99%; numbers on short branches were removed for clarity. **B.** Map of the Galápagos archipelago showing islands included in this study. The approximate location of Mares Islet is indicated with an arrow. This map was created using the Free and Open Source QGIS; base map and data from OpenStreetMap and OpenStreetMap Foundation. **C.** *Phyllodactylus maresi* from Rábida (photograph by Fernando Ayala-Varela, Bioweb.bio).

The topologies of the maximum likelihood and Bayesian trees obtained in this study (Fig 1 and S1 Fig, respectively) are very similar and support previous hypotheses [8,18]. All species of *Phyllodactylus*, except for *P. darwini* and *P. gilberti* are nested in a single clade, the “*galapagensis* clade” [6]. The new specimens of *Phyllodactylus* from Rábida form a strongly supported clade sister to *P. maresi* from Santiago and Marchena with maximum support. Therefore, we used all available samples from Rábida, Santiago and Marchena in the phylogeographic analyses.

**Fig 2:**
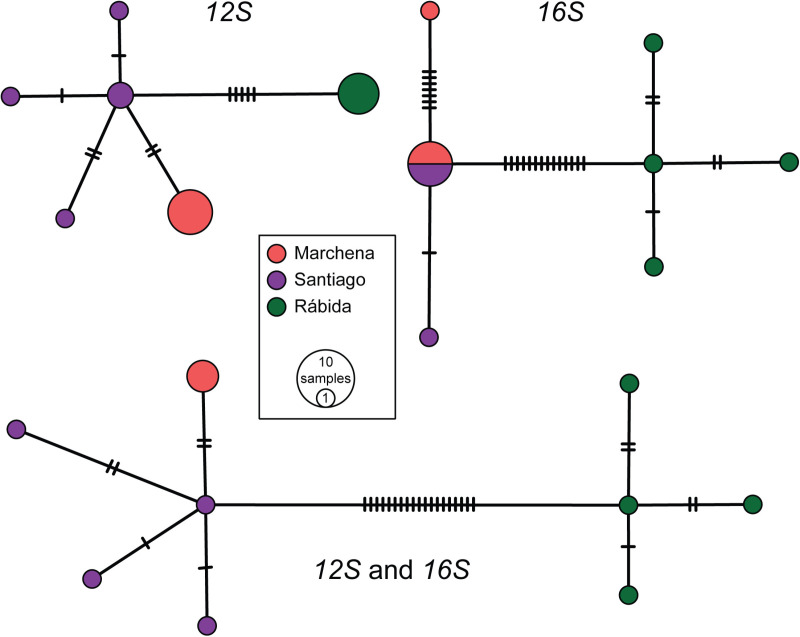
Haplotype networks of leaf-toed geckos (*Phyllodactylus*) from Rábida, Santiago and Marchena islands. The haplotype networks are based on both individual *12S* and *16S* genes and a concatenated dataset. Bars on branches indicate mutation steps. The colors match the clades in Fig 1.

The inferred networks show a close genetic similarity between populations from Marchena and Santiago; however, samples from Rábida are more divergent (Fig 2). The concatenated *12S-16S* network shows four haplotypes from Santiago separated from each other by 1–2 mutations, one haplotype from Marchena separated by two mutations from Santiago, and four haplotypes from Rábida separated from each other by 1–2 mutations and from Santiago by 19 mutations. The *12S*-only network is different in that Rábida contains a single haplotype, separated from Santiago by four mutations. The *16S*-only network shows a shared haplotype between Santiago and Marchena, separated from single haplotypes from Santiago and Marchena by one and seven mutations, respectively; haplotypes from Rábida are separated from the shared Santiago/Marchena haplotype by 14 mutations.

Mean *12S* and *16S* interspecific genetic distances (S1 and S2 Tables, respectively) among species of the *P. galapagensis* radiation vary between 0.02 *(P. duncanensis*/ *P. simpsoni*) and 0.11 (e.g., *P. maresi*/ *P. leei*, *P. gorii*/ *P. baurii*), and 0.01 (*P. simpsoni*/ *P. andysabini*) and 0.10 (e.g., *P. duncanensis/ P. leei*, *P. galapagensis*/ *P. gorii*), respectively. The mean *12S* genetic distance is 0.01 between specimens of *P. maresi* from Santiago and Marchena, 0.01 between Santiago and Rábida, and 0.02 between Marchena and Rábida. The mean *16S* genetic distance is 0.002 between specimens of *P. maresi* from Santiago and Marchena, 0.01 between Santiago and Rábida, and 0.01 between Marchena and Rábida.

The specimens of *Phyllodactylus* from Rábida are similar in morphology and color pattern to those from Santiago and Mares islet (Table 2). Specimens from Rábida are more similar to those from Santiago in having less paravertebral tubercles and less scales around midbody. Given the lack of DNA samples from Mares, lack of specimens from Marchena, and generally low sample sizes, we assign the samples of leaf-toed geckos from Rábida to *P. maresi* based on our phylogenetic analysis (Fig 1).

**Table 2:** Geographical variation in morphology of *Phyllodactylus maresi* from Mares, Santiago and Rábida islands.

Character	Mares n = 5	Santiago n = 3	Rábida n = 10
Scales from nostril to eye	11–13 12.00 ± 0.71	11–12 11.33 ± 0.58	11–13 11.80 ± 0.79
**Scales across snout at level of third labials**	**18–21** **19.00 ± 1.41**	**22–24** **22.67 ± 1.15**	**18–26** **23.10 ± 2.42**
Interorbitals at level of center of eyes	24–27 25.40 ± 1.14	23–27 24.33 ± 2.31	15–28 22.00 ± 4.27
Supralabials to a point below center of eye	7–9 8.00 ± 0.71	7–8 n = 2	6–7 6.60 ± 0.52
Infralabials to a point below center of eye	6–7 6.60 ± 0.55	6	5–6 5.70 ± 0.48
Postmentals	2–4 3.00 ± 1.00	2–4 3.00 ± 1.00	3–4 3.50 ± 0.53
Scales in contact with postmentals posteriorly	7–8 7.40 ± 0.55	7	7–9 7.80 ± 0.79
Scales in contact with scales behind postmentals	9–12 10.60 ± 1.14	9–11 10.00 ± 1.00	9–12 10.70 ± 1.16
Longitudinal rows of dorsal tubercles	12–13 12.20 ± 0.45	12	10–12 11.70 ± 0.67
**Paravertebral tubercles from head to base of tail**	**44–53** **50.20 ± 3.63**	**39–42** **40.67 ± 1.53**	**33–45** **38.80 ± 3.91**
**Paravertebral tubercles from axilla to groin**	**30–38** **33.40 ± 3.21**	**24–27** **25.00 ± 1.73**	**23–28** **25.80 ± 1.62**
**Scales around midbody**	**82–94** **86.60 ± 4.93**	**77–78** **77.50 ± 0.71**	**60–79** **71.90 ± 6.77**
Lamellae on Finger IV	10–11 10.80 ± 0.45	9 n = 1	7–10 8.90 ± 0.74
Lamellae on Toe IV	11–12 11.60 ± 0.55	11 n = 1	7–11 9.80 ± 1.03
Maximum SVL in mm	43	34	41
Eye diameter in mm	3.1–3.4 3.22 ± 0.11	2–2.6 2.23 ± 0.32	2.4–3.3 2.86 ± 0.28
Head length/ SVL	0.26–0.28 0.28 ± 0.01	0.25–0.33 0.28 ± 0.04	0.25–0.35 0.28 ± 0.03
Head length/ head width	1.42–1.60 1.51 ± 0.07	1.33–1.64 1.45 ± 0.17	1.23–1.58 1.42 ± 0.10
Snout length/ SVL	0.13–0.14 0.14 ± 0.00	0.12–0.15 0.14 ± 0.02	0.07–0.13 0.09 ± 0.02
Axilla-groin length/ SVL	0.39–0.46 0.42 ± 0.03	0.44–0.47 0.45 ± 0.02	0.35–0.50 0.39 ± 0.04

Data for populations from Mares and Santiago was taken from Lanza [11]. Range (first line), mean ± SD (second line), and n (if different from heading, third line) are given. We subtracted “2” from both finger and toe lamellae counts reported by Lanza [11] because he explicitly counted the adhesive scansors (two on each digit) as lamellae. Characters noted as different between populations from Mares and Santiago by Lanza [11] are in bold.

## Discussion

The Galápagos Archipelago represents an iconic conservation site. Therefore, it is crucial for conservation practice that biodiversity data is based on evidence instead of anecdote [31], and that conservation interventions (e.g., removal of invasive species) have a robust monitoring program [32] that can provide attribution of changes detected to specific interventions. This may include changes in density, distribution, recording new species or rediscovering others. The recent discovery of new species of land iguanas, giant tortoises, snakes, and lizards [8,33–35], as well as the rediscovery of species that were thought to be extinct [5], underscores the fact that the task of documenting the biodiversity of terrestrial vertebrates in the Galápagos is far from complete. The best scenario to overcome this problem in common species like lava lizards and leaf-toed geckos is to collect whole-organism voucher specimens with associated DNA samples in areas determined by expert taxonomists. Except for endangered species or populations, whole organism specimens are essential for species descriptions and conservation actions [36]. Although leaf-toed geckos have been collected in many islands across the Galápagos archipelago [7,9–11], records from Rábida have only been anecdotal [14,16], except for subfossils [13] and a photograph of a specimen recorded in 2012 [15]. The 2012 specimen, although collected, does not appear in collections where it was reported as deposited and now appears to be lost. Here we confirm the existence of *Phyllodactylus* in Rábida through collection of voucher specimens as part of the monitoring program following the 2011 invasive rodent eradication program. Teams (including authors KC, WC) searching for geckos were unable to find geckos or any evidence of them (e.g., footprints, shed skin, egg shells) during and prior to 2011. Lizard populations subject to rodent predation in the Caribbean and offshore islands of New Zealand have recovered after rodent eradication [37,38]. Although the ecological relationships between rodents and geckos in Rábida are unknown, we suggest that populations of *Phyllodactylus* benefited from the eradication of rodents in Rábida and that population recovery and expanded distribution due to rodent eradication facilitated their rediscovery and the collection efforts reported here.

Our phylogenetic analyses suggest that the population of *Phyllodactylus* from Rábida corresponds to *P. maresi*, a species that was recently elevated from subspecies status by Arteaga *et al.* [16]. Although we accept *P. maresi* as a valid species, we argue that Arteaga *et al.*‘s proposal is questionable because they did not include in their analyses samples from Mares islet (Fig 1), the type locality of *P. maresi* [11]. In a comprehensive phylogenetic study of *Phyllodactylus* from the Galápagos, Torres-Carvajal *et al.* [8] recognized a clade with six samples from Santiago and Marchena as “*P*. sp. 2”. Upon addition of mitochondrial gene sequences of three samples from Marchena, Arteaga *et al.* [16] obtained a similar topology and assigned arbitrarily populations from Marchena, Santiago and the surrounding Rábida, Bartolomé and Mares Islet to *Phyllodactylus maresi*. However, it is worth mentioning that Lanza [11] proposed the subspecies *P. galapagensis maresi* precisely because he noticed morphological differences between specimens from Mares and Santiago. We found similar differences between specimens from Rábida and Mares (Table 2). Strikingly, there are only five whole-body voucher specimens of *Phyllodactylus* from Mares, three from Santiago and no specimens from Marchena in museums worldwide as far as we know. Therefore, further and possibly more refined (e.g., genomic data) analyses based on additional sampling is needed to elucidate the taxonomic status of leaf-toed geckos from Marchena, Rábida, Santiago, Mares and surrounding islets. Phylogenomic analyses are particularly promising as demonstrated by a previous study on *Phyllodactylus* geckos from Mexico that revealed substantial cryptic diversity [39].

The delimitation of evolutionarily significant units (ESUs)—divergent intraspecific lineages [40]—is crucial for the conservation of insular species, which are represented by relatively small and isolated populations that are generally more prone to extinction than their mainland relatives partly because of limited genetic variation [41]. Genetic variability in *Phyllodactylus* is lower in the Galápagos than in mainland South America [8]; therefore, recognition of ESUs are important for the conservation of these lizards. Torres-Carvajal *et al.* [42] recognized two ESUs within *P. baurii*, one occurring in Floreana and Champion, and the other from Gardner. Despite taxonomic uncertainty, both our phylogenetic and phylogeographic analyses suggest that leaf-toed geckos from Rábida represent a separate ESU from the Marchena and Santiago populations (Figs 1 and 2).

## Supporting information

S1 Table *12S* uncorrected pairwise distances among species of the *Phyllodactylus galapagensis* radiation. Number of sequences (N) of each taxon, mean, standard error, minimum (Min), Q1, median, Q3, and maximum (Max) values are presented.
(XLSX)

S2 Table *16S* uncorrected pairwise distances among species of the *Phyllodactylus galapagensis* radiation. Number of sequences (N) of each taxon, mean, standard error, minimum (Min), Q1, median, Q3, and maximum (Max) values are presented.
(XLSX)

S1 Fig Bayesian phylogenetic tree of leaf-toed geckos (*Phyllodactylus*). Numbers on branches are posterior probability values.
(PDF)

S2 Fig Maximum-likelihood phylogenetic tree of leaf-toed geckos (*Phyllodactylus*). Numbers on branches are SH-aLRT/ultrafast bootstrap support values.
(PDF)
