# Supplementary figures and images for: Phylogeny, phylogeography, and conservation of a rediscovered gecko from the Galápagos Islands
Source: PLoS One. 2025 Jun 13;20(6):e0324659. doi: 10.1371/journal.pone.0324659 (PMC12165420; doi:10.1371/journal.pone.0324659)

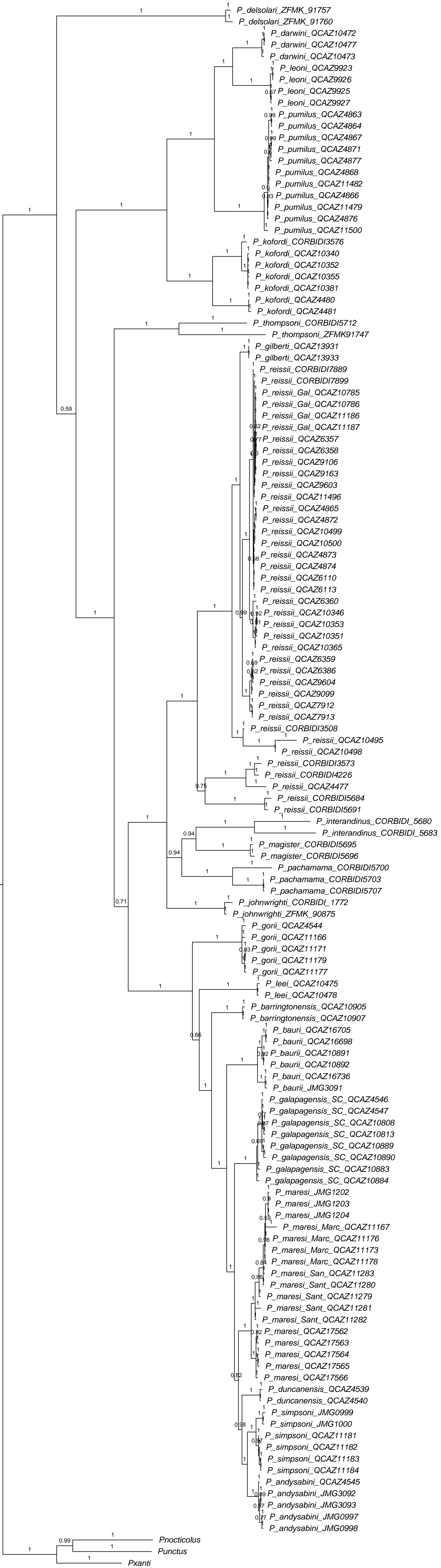

Supplement: S1 Fig — Numbers on branches are posterior probability values. (PDF) [file pone.0324659.s003.pdf]

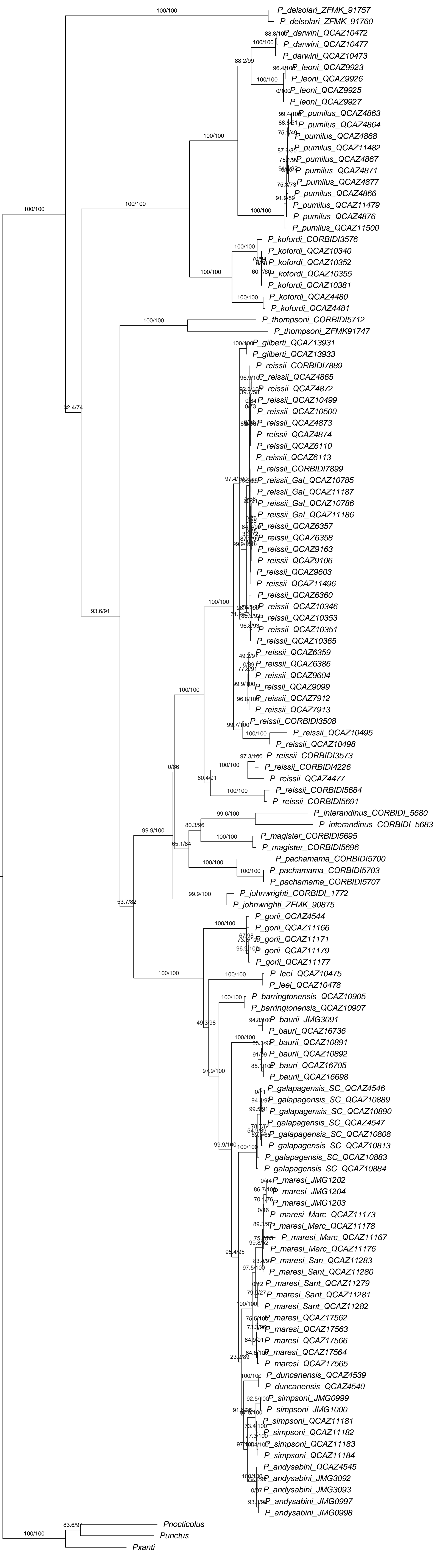

Supplement: S2 Fig — Numbers on branches are SH-aLRT/ultrafast bootstrap support values. (PDF) [file pone.0324659.s004.pdf]
